# The reliability of a portable clinical force plate used for the assessment of static postural control: repeated measures reliability study

**DOI:** 10.1186/2045-709X-20-14

**Published:** 2012-05-23

**Authors:** Samira Golriz, Jeffrey J Hebert, K Bo Foreman, Bruce F Walker

**Affiliations:** 1 School of Chiropractic and Sports Science, Murdoch University, 90 South Street, Murdoch, WA, 6150, Australia; 2 School of Chiropractic and Sports Science, Murdoch University, Perth, WA, Australia; 3 Department of Physical Therapy, University of Utah, Salt Lake City, UT, USA

**Keywords:** Reproducibility, Posture, Stability, Balance, Force plate

## Abstract

**Background:**

Force plates are frequently used for postural control assessments but they are expensive and not widely available in most clinical settings. Increasingly, clinicians are using this technology to assess patients, however, the psychometric properties of these less sophisticated force plates is frequently unknown. The purposes of the study were to examine the test-retest reliability of a force plate commonly used by clinicians and to explore the effect of using the mean value from multiple repetitions on reliability.

**Methods:**

Thirty healthy volunteer adults were recruited. Postural control measures were obtained using the Midot Posture Scale Analyzer (MPSA). Data were collected in 2 sessions. Five successive repetitions each of 60 seconds duration were obtained from each participant in each session.

**Results:**

The reliability coefficients obtained using single measures were low (ICC_3,1_ = 0.06 to 0.53). The average of two measures allowed for reliable measurements of COP mean velocity and average location of COP. The average of three and five measures was required to obtain acceptable reliability (ICC ≥ 0.70) of relative weight bearing on legs and sway area, respectively. Higher measurement precision values were seen by averaging four or five repetitions for all variables.

**Conclusion:**

Single measures did not provide reliable estimates of postural sway, and the averaging of multiple repetitions was necessary to achieve acceptable levels of measurement error. The number of repetitions required to achieve reliable data ranged from 2 to 5. Clinicians should be wary of using single measures derived from similar equipment when making decisions about patients.

## Introduction

Postural control organises the orientation and equilibrium of the body during upright stance and is essential to the successful performance of daily movements and activities as well as fall prevention [1]. Postural control depends on visual, vestibular and proprioceptive input and can be disrupted by various perturbations experienced in everyday life [2,3]. Moreover, pathology, medications, alcohol consumption, and the aging process can adversely affect postural control [4,5] .

Postural control can be measured subjectively or objectively. Subjective measures of postural control are obtained through the use of questionnaires. Such questionnaires provide valuable information, however they often have limitations with some special populations such as the elderly or individuals with specific physical or cognitive impairment [6,7]. In addition, subjective methods of measurement may suffer from floor or ceiling effects or lack optimal reliability, validity and the precision to detect small differences [8,9]. It has been suggested that these questionnaires be used in combination with other measures [9].

Objective assessments are the most common method of measuring postural control. Postural control is usually evaluated by interpretation of centre of pressure (COP), postural sway and weight bearing distribution measurements [10,11]. COP estimates the position of the ground reaction force vector on the base of support of the body and calculation of the total COP kinematics is frequently used to assess postural sway [12]. Postural sway is an indicator of the displacement and correction of the centre of gravity in relation to the base of support [11]. Relative weight bearing is a gross estimate of postural control. Postural control is influenced by weight bearing distribution and weight bearing distribution asymmetry results in less postural stability [10].

Force plates are frequently used to measure COP and postural sway [11]. This approach requires the individual to stand or walk, while transducers measure ground reaction forces generated by the body. As a clinical tool, force plates are utilised therapeutically [13], and for longitudinal assessment [14]. Force plates can be used to enhance balance training by providing visual feedback to the patient [13]. However, the types of force plates used by clinicians typically lack the sophistication of force plates used in research environments. Moreover, well known force plates used in research have known psychometric properties such as reliability and validity, while the psychometric properties of force plates used in the clinical setting are frequently unknown or poorly defined.

The Midot posture scale analyser (MPSA) is an example of less sophisticated force plate used by clinicians such as physical therapists, chiropractors, and neurologists [15]. The MPSA is relatively inexpensive and includes simple and easy to use software that allows individuals with limited experience to obtain and interpret measures of postural control and weight bearing distribution.

However, when making decisions about patients, it is logical to rely on assessments obtained by instruments shown to be reliable and valid. Reliability is a prerequisite of validity and reflects measurement consistency and the degree to which an instrument is free from errors of measurement [16]. When clinicians obtain measurements to inform clinical decision making, it is necessary that the instruments demonstrate adequate reliability. Therefore, the purpose of this study was to examine the test-retest reliability of a force plate commonly used by clinicians. Additionally, we explored the effect of using the mean value from multiple repetitions on reliability.

## Methods

### Participants

Participant recruitment was by way of posted advertisements on University bulletin boards. Potential participants were adults between 18 to 60 years of age. A lower age limit of 18 years was chosen to help ensure maturity of the skeletal system [17]. Postural control decreases in the elderly [18], therefore 60 years was considered the upper age limit.

Participants were excluded from the study if they had a history of musculoskeletal injury in the previous three months, a balance deficit stemming from a rheumatologic or neurologic disorder, pregnancy, an ear infection or fever within 72 hours of testing or were currently taking medications that could alter sensory perception. The Human Research Ethics Committee of Murdoch University approved the study protocol (2010/139), and all subjects gave written consent before enrolling in the study. The rights of all subjects were protected.

### Instrument

The Midot posture scale analyser (MPSA)- QPS 200 is a portable force plate consisting of 4 electronic weighing plates set in a rectangular position. Analogue signals were sampled and transferred to a laptop computer via a USB-to-serial (RS-232) analogue to digital converter. The MPSA specifications report an acquisition sampling frequency of 200 Hz and cut-off frequency of 0.5 Hz [15].

The MPSA is designed to measure, during quiet standing: (1) the average location of COP with reference to the cross point of the weighing platform (mm), (2) COP mean velocity, which is the average velocity of COP movement (mm/sec), (3) sway area, which is the area of an ellipse enclosing 95% of COP movement (mm^2^) and was measured by calculation of the ellipse area, and (4) the relative weight distribution between the subject’s right and left sides (%).

### Procedure

Calibration of the force plate was conducted each day prior to data collection based on the manufacturer’s instructions. We tested all subjects under the same conditions. Participants stood with their feet shoulder width apart on a sheet of paper placed on the top of the platform. The paper remained in place during testing. Both feet were outlined to ensure consistent placement across trials. Participants were asked to remove their shoes, stand upright on the force plate and remain as still as possible in a relaxed posture. We asked participants to put their arms to their sides in a comfortable position and to distribute their body weight evenly on both feet while breathing normally. Finally, the participants were instructed to look straight ahead at an “X” on the opposite wall located 2 meters away at eye level. If the patient usually wore glasses, they continued to do so during this procedure.

Subjects were scheduled for 2 sessions of 5 trials, 5 minutes apart. The second session replicated the first. The duration of each trial was 60 seconds. A 60-second assessment was chosen to mimic constituent periods of standing during typical activities of daily living (e.g., waiting for a bus or elevator). To avoid inconsistencies in the data at transitions, we informed the participants of the data collection start time 5 seconds before the actual start time. Mandatory breaks of 1 minute were allocated between each individual trial during which subjects were allowed to sit. Breaks assured that participants were refreshed for each trial. Five successive trials were recorded during each session. On average, the overall duration of the experiment was approximately 25 minutes.

### Statistical analysis

Using the approach of Donner and Eliasziw (1987) [19], considering a minimally acceptable intraclass correlation coefficient (ICC) value of 0.70 [20] and 5 repetitions in each of two sessions, recruitment of 30 participants at an alpha level of 0.05 was estimated to provide 80% power to detect a relationship this strong or stronger.

Data management and statistical analyses were performed using SPSS version 17. Data were entered and inspected to ensure that there were no errors of entry. Descriptive statistics were calculated for all variables.

Values for a single repetition and averages of 2, 3, 4 and 5 repetitions were calculated for all dependant variables. To visually examine for the presence of systematic error (e. g., fatigue or learning effects) average values of COP mean velocity and sway area were plotted against the number of repetitions. Relative reliability of the measures was assessed using ICC_3,k_[21] and 95% confidence intervals. The standard error of measurement was calculated to assess measurement precision using the formula: $\text{Standard error of measurement}=\text{pooled standard deviation}\times \sqrt{1-\text{ICC}}\text{.}$ Additionally, we calculated the minimal detectable difference (MDD) with 95% confidence intervals. The MDD was calculated using the formula: $\text{MDD}=\;\text{1.96}\times \text{standard error of }\text{measurement}\times \sqrt{2}$. and estimates the smallest difference exceeding measurement error [16]. To explore for systematic differences between the first and second sessions, bias statistics with 95% confidence intervals were calculated by computing the mean difference of measures obtained during the two sessions. Levels of agreement (LOA) were calculated as: LOA = bias ±1.96standard deviation of differences between the2sessions. It is expected that 95% of the difference between the first and second sessions would be between these limits [22]. Finally, to compare the effect of the number of repetitions on reliability, all of the above calculations were generated using values from a single repetition as well as the mean of the first 2, 3, 4 and 5 repetitions.

## Results

Thirty volunteers aged 20 to 57 years participated in the study. All subjects were able to complete the protocol and all data were included for statistical analysis. The final sample was composed of 16 men and 14 women, with a mean ± SD age of 30.5 ± 7.2 years and BMI of 25.6 ± 5.5.

The profile plots displaying the averages of COP mean velocity and sway area across repetitions were visually inspected and no learning or fatigue effects were identified. Means, standard deviations, reliability coefficients, standard error of measurement, MDD, bias and LOA statistics are presented in Table 1 for each dependant variable.

**Table 1:** Reliability results of single measures and means of 2, 3, 4 and 5 measures for each variable

**Variable**	Mean ± SD^*^	**ICC (95% CI)**	**SEM**	**MDD**	**Bias (95% CI) ± 95% LOA**
**Relative weight bearing on right leg (%)**					
1 repetition	50.10 ± 2.7	0.44 (−0.18,0.74)	2.0	5.6	0.3(−0.3,0.9) ±6.5
2 repetitions	49.84 ± 2.4	0.65 (0.25,0.83)	1.4	3.9	0.3(−0.2,0.7) ±4.9
3 repetitions	49.69 ± 2.3	**0.75** (0.47,0.88)	1.2	3.2	0.1(−0.3,0.5) ±4.1
4 repetitions	49.69 ± 2.3	**0.82** (0.63,0.92)	1.0	2.7	0.2(−0.2,0.5) ±3.6
5 repetitions	49.64 ± 2.3	**0.83** (0.64,0.92)	0.9	2.6	0.3(−0.1,0.6) ±3.5
**Relative weight bearing on left leg (%)**					
1 repetition	49.90 ± 2.7	0.44(−0.18,0.75)	2.0	5.6	0.3(−0.3,0.9) ±6.5
2 repetitions	50.16 ± 2.4	0.65 (0.27,0.85)	1.4	3.9	0.3 (−0.2,0.7) ±4.9
3 repetitions	50.31 ± 2.3	**0.75** (0.49,0.89)	1.2	3.2	0.1(−0.3,0.5) ±4.1
4 repetitions	50.31 ± 2.3	**0.81** (0.59-0.91)	1.0	2.7	0.1(−0.2,0.5) ± 3.7
5 repetitions	50.36 ± 2.3	**0.82** (0.63-0.92)	1.0	2.7	0.2(−0.1,0.5) ±3.5
**COP mean velocity (mm/sec)**					
1 repetition	6.0 ± 4.8	0.19 (−0.75,0.62)	4.4	12.1	−0.6(−1.9,0.7) ±13.50
2 repetitions	5.6 ± 3.4	**0.83** (0.65,0.92)	1.3	3.5	0.2(0.1,0.4)† ±1.81
3 repetitions	5.5 ±3.3	**0.95** (0.90,0.98)	0.7	2	0.2(0.0,0.5) ±2.68
4 repetitions	5.5 ± 3.3	**0.97** (0.94,0.99)	0.6	1.6	0.09(−0.1,0.3) ±2.09
5 repetitions	5.4 ± 3.2	**0.92** (0.84,0.96)	0.9	2.5	0.03(−0.3,0.3) ±3.43
**Average location of COP (mm)**					
1 repetition	35.0 ± 21	0.53 (−0.01,0.78)	14.4	39.7	−1.8(−6.2,2.6) ±47.2
2 repetitions	38.4 ± 31.1	**0.92** (0.84,0.96)	8.8	24.4	−1.6(−4.7,1.5) ±33.3
3 repetitions	37.3 ± 26.5	**0.91** (0.81,0.96)	8	22.1	−0.4(−3.2,2.4) ±30.2
4 repetitions	38.8 ± 28.9	**0.93** (0.85,0.97)	7.7	21.3	−0.7(−3.4,2.1) ±29.8
5 repetitions	39.0 ± 29.6	**0.94** (0.88,0.97)	7.3	22	−0.6(−3.2,2.0) ±27.9
**Sway area (mm**^**2**^**)**					
1 repetition	1549.6 ± 1605.5	0.06(−1.02,0.56)	1556.9	4302.7	−265.6(−686.4,155.1) ± 4517.0
2 repetitions	1442.6 ± 1055.5	0.47 (−0.13,0.75)	763.7	2116.8	−73.2 (−302.3,156.3) ±1399.0
3 repetitions	1456.6 ± 1001.9	0.63 (0.28,0.82)	612.7	1698.3	−159.2 (−350.4,32.1) ±2054.0
4 repetitions	1440.3 ± 889.8	0.68 (0.33,0.85)	504.8	1399.2	−159.2 (−278.9,43.8) ± 1732.7
5 repetitions	1466.9 ± 918.2	**0.83** (0.64,0.92)	380.4	1054.3	−48.6 (−178.7,81.7) ± 1399.0

*CI* confidence interval, *ICC* intra class correlation coefficient, *LOA* limit of agreement, *MDD* minimal detectable difference, *SEM* standard error of measurement

*Pooled from all repetitions;

† Statistically significant bias (different from zero);

Bold ICCs indicate acceptable reliability values.

Each of the five variables measured exhibited unacceptable levels of measurement error when calculated from single measures. For the relative weight distribution on the legs, it was necessary to average 3 or more repetitions to achieve a minimum acceptable reliability value. COP mean velocity required at least 2 trials to obtain an acceptable ICC value. The ICC values of the average COP location are acceptable with 2 measurements; while measures of sway area required 5 repetitions to achieve acceptable reliability.

Bias estimates were small and not significantly different from zero indicating that there were no statistically significant differences between the two sessions. The only exception was the COP mean velocity calculated of two repetitions, which reached the threshold of statistical significance, however the magnitude of this difference was small.

## Discussion

The objectives of this study were to examine the test-retest reliability of postural control measurements of a portable force plate commonly used by clinicians and to explore the effect of using the mean value from multiple repetitions on reliability. Our results demonstrated that for measures obtained by the MPSA, single trials do not provide reliable estimates of postural control and that averaging multiple measures is necessary to achieve acceptable levels of measurement error. The number of repetitions necessary to achieve reliable results varied depending of the outcome variable and ranged from two to five. Clinicians should take this into account when measuring postural control on their patients.

ICC values for measures of relative weight bearing appeared lower than ICC values of COP mean velocity and average location of COP. However, inspection of the descriptive statistics (mean and SD) in Table 1 for each of these variables indicates less inter-subject variability in relative weight bearing as compared to the other dependent variables. Low levels of inter-subject variability are known to artificially lower ICC estimates, as this increases the relative magnitude of the error term in the ICC equation [16]. Thus, it can be difficult to interpret ICC values derived from homogenous measures such as relative weight distribution.

We also assessed COP mean velocity, average location of COP, and sway area. Consistent with our results, COP mean velocity has been reported by others to be the most reliable estimate of COP [23-25]. In contrast, others have examined samples of healthy participants and reported low reliability for measures of COP mean velocity [26]. However, it should be noted that in that study, the ICC statistics were calculated by averaging data from 3 10-second repetitions. The longer duration of recording used in our protocol may explain our higher reliability estimates. While longer duration trials of up to 120 seconds are recommended to reduce measurement error [24], sampling duration should be matched to the abilities of participants. For instance, children with cerebral palsy or the elderly may not tolerate standing for an ideal duration of time.

For measures of sway area, it was necessary to average values from five repetitions to achieve an acceptable level of measurement error. Two studies reported similarly low ICC values for sway area [27,28]. Alternatively, others have reported acceptable levels of ICC for this variable; however, these latter studies were conducted under eyes closed.

A potential issue with relying on measures obtained from a suboptimal number of repetitions can arise in clinical practice. For instance, when examining for differences in postural stability over using single measures, a practitioner needs to observe an improvement of at least 12.1 mm/sec in COP mean velocity to be 95% confident that a true change has occurred. However, when using a mean of 3 repetitions, a practitioner can be just as confident that true change has occurred with a change of 2 mm/sec.

The results of this study are limited by several factors. This study was conducted on healthy individuals and the results may or may not generalize to clinical populations. Moreover, the MPSA as an instrument of measurement has several limitations. The MPSA is limited to a fixed duration of data acquisition of between 5 and 60 seconds. It is not possible to set up the recording time to durations longer than 60 seconds, which may be desirable is some circumstances. This technology also has a fixed sampling rate and cut-off frequency and altering these frequencies is not possible. Finally, the MPSA software does not report some variables such as sway area, which we calculated from the raw data.

Future research should examine the validity of the force plates commonly used by clinicians by comparing their measures to those obtained using force plates with known validity. Additionally, it would be useful to assess the reliability of similar force plates in a clinical population, such as those individuals with neurological impairments.

## Conclusion

For measures of postural sway obtained by the MPSA, single trials do not provide reliable estimates, and the averaging of multiple repetitions was necessary to achieve acceptable levels of measurement error. Depending on the variable, the number of repetitions required to achieve reliable data ranged from 2 to 5. Clinicians should be wary of using single measures derived from similar equipment when making decisions about patients.

## Abbreviations

COP, Centre of pressure; ICC, Intraclass correlation coefficient; LOA, Level of agreement; MDD, Minimal detectable difference; MPSA, Midot posture scale analyser.

## Competing interests

The authors declare that they have no competing interests.

## Authors’ contributions

SG participated in the design of the study, collected data, performed the statistical analysis and drafted the manuscript. JH participated in the design of the study, helped to perform the statistical analysis and drafted the manuscript. BF helped to draft the manuscript. BW participated in the design of the study and helped to draft the manuscript. All authors read and approved the final manuscript.
